# Growth responses of *Ulva prolifera* to inorganic and organic nutrients: Implications for macroalgal blooms in the southern Yellow Sea, China

**DOI:** 10.1038/srep26498

**Published:** 2016-05-20

**Authors:** Hongmei Li, Yongyu Zhang, Xiurong Han, Xiaoyong Shi, Richard B. Rivkin, Louis Legendre

**Affiliations:** 1 Research Center for Marine Biology and Carbon Sequestration, Shandong Provincial Key Laboratory of Energy Genetics, Qingdao Institute of Bioenergy and Bioprocess Technology, Chinese Academy of Sciences, Qingdao, 266101, China; 2 College of Chemistry and Chemical Engineering, Ocean University of China, Qingdao, 266100, China; 3 National Marine Hazard Mitigation Service, Beijing, 100194, China; 4 Department of Ocean Sciences, Memorial University of Newfoundland, St. John’s, NL A1C 5S7, Canada; 5 Sorbonne Universités, UPMC Université Paris 06, CNRS, Laboratoire d’océanographie de Villefranche (LOV), Observatoire océanologique, 181 Chemin du Lazaret, 06230 Villefranche-sur-Mer, France

## Abstract

The marine macrophyte *Ulva prolifera* is the dominant green-tide-forming seaweed in the southern Yellow Sea, China. Here we assessed, in the laboratory, the growth rate and nutrient uptake responses of *U. prolifera* to different nutrient treatments. The growth rates were enhanced in incubations with added organic and inorganic nitrogen [i.e. nitrate (NO_3_^−^), ammonium (NH_4_^+^), urea and glycine] and phosphorus [i.e. phosphate (PO_4_^3−^), adenosine triphosphate (ATP) and glucose 6-phosphate (G-6-P)], relative to the control. The relative growth rates of *U. prolifera* were higher when enriched with dissolved organic nitrogen (urea and glycine) and phosphorus (ATP and G-6-P) than inorganic nitrogen (NO_3_^−^ and NH_4_^+^) and phosphorus (PO_4_^3−^). In contrast, the affinity was higher for inorganic than organic nutrients. Field data in the southern Yellow Sea showed significant inverse correlations between macroalgal biomass and dissolved organic nutrients. Our laboratory and field results indicated that organic nutrients such as urea, glycine and ATP, may contribute to the development of macroalgal blooms in the southern Yellow Sea.

Anthropogenic activities, including rapidly increasing population, excessive agricultural fertilization, and intensive mariculture, have caused increasing inputs of inorganic and organic nutrients into coastal waters, which contribute to eutrophication and harmful algal blooms (HABs)^1^,^2^. Green tides, a type of HAB that can be caused by accumulation of free-floating macroalgae, have been persistent features in eutrophic coastal waters and in estuaries worldwide^3^,^4^. Large blooms of the green alga *U. prolifera* occurred yearly in the southern Yellow Sea, China, from 2007 to 2015. They were among the largest of such outbreaks worldwide, and the large scale accumulations of macroalgae biomass caused severe environmental problems in the southern Yellow Sea^5^,^6^.

Macroalgal blooms in the southern Yellow Sea generally originate from small patches of floating green algae in coastal areas of the Jiangsu Province during mid-April to early May. These algae drift northward in the southern Yellow Sea for about 1.5 months and reach the coast near Qingdao in July or August (Supplementary Fig. S1)^1^,^7^. Previous studies on the spatiotemporal variations of dissolved inorganic and organic nutrients in the southern Yellow Sea during the period of macroalgal blooms in 2012 inferred that, although the concentrations of inorganic N and P ranged from 0.2 to 36.5 and 0 to 0.6 μM, respectively, dissolved organic nutrients may be taken up and contribute to the development and persistence of macroalgal blooms^6^. Indeed it was reported that, during the development of a macroalgal bloom in the southern Yellow Sea, there was an increase in the biomass of *U. prolifera* and area it covered and a decline in the concentrations of organic N and P^6^,^8^. It was also suggested that during the mid- to late period of the macroalgal blooms, *U. prolifera* almost exhausted dissolved inorganic N- and P-nutrients, and continued growth may have been supported by organic nutrients^9^ and remineralized forms of N and P.

Macroalgal blooms in coastal waters are often the result of nutrient eutrophication, such as increased nutrient loads in river runoff and inputs from mariculture^5^,^10^. In the southern Yellow Sea, the Sheyang (33.7 °N) and Guanhe (34.3 °N) rivers discharge large quantities of inorganic and organic nutrients into the Jiangsu coastal waters, thus contributing to coastal eutrophication^11^. Marine aquaculture, particularly shrimp and shellfish, expanded rapidly in the Jiangsu Province during the last decade, and the total area for aquaculture along the southern Yellow Sea coast has increased to 201 kha in 2011, doubling the annual production (8.42 × 10^5 ^t) relative to 2003 (4.10 × 10^5 ^t)^12^. In addition, ~50,000 t of fertilizers rich in organic N and P, such as fermented chicken manure, were added annually to land-based aquaculture ponds along the Jiangsu Coast^5^. The discharge of nutrient-rich wastewater from these aquaculture ponds could also supply organic and inorganic nutrients to nearshore coastal waters.

Previous studies have shown that *U. prolifera* is the most dominant green-tide-forming species in the southern Yellow Sea^13^. The fronds of *U. prolifera* generally exhibit high rates of nutrient uptake and growth in eutrophic waters, and are tolerant to changes in temperature, salinity, light and desiccation^14^. These characteristics provide a competitive advantage to *U. prolifera*, relative to other macroalgae in the eutrophic coastal waters of the southern Yellow Sea^15^. Moreover, laboratory experiments show that *U. prolifera* rapidly assimilates dissolved inorganic N and P^16^,^17^, with a higher uptake rate per unit biomass of N than P^16^. Some field studies in the southern Yellow Sea have suggested that dissolved organic N and P may also be used by *U. prolifera* for growth^5^,^6^,^18^. Although the uptake of organic nutrients by other macroalgae has been studied and is well understood^19^,^20^, the uptake of organic N and P by *U. prolifera* has seldom been investigated in the laboratory^21^.

Planktonic algae take up NO_3_^−^ and NH_4_^+^ 22 and as well as some forms of organic N^23^. Macroalgae take up inorganic nitrogen, urea and some free amino acids^20^,^21^. Urea is present in coastal marine waters, especially in regions impacted by anthropogenic activities, where it can account for ~5% of the dissolved N in the southern Yellow Sea during the spring of 2012^9^. Dissolved free amino acids (DFAA), such as glycine, serine and alanine, are also common forms of organic N in seawater^24^. The major form of dissolved inorganic P used by algae is PO_4_^3−^. Organic P can be as important as orthophosphate in supporting the growth of dinoflagellates^25^ and other marine phytoplankton^26^ when ambient PO_4_^3−^ concentrations are low^25^,^26^. Adenosine triphosphate (ATP) is a low-molecular-weight form of organic P in seawater and concentrations of dissolved ATP range from 0.1 to 0.6 μg L^−1^ 27,28. Another form of low molecular weight organic-P that is rapidly cycled in algal cells is glucose 6-phosphate (G-6-P), and although it has not been measured in seawater, it is assimilated by marine phytoplankton^26^,^29^. Since ATP and G-6-P either occur in seawater or have been shown to be taken up by microalgae^26^,^30^,^31^,^32^, we used them for macroalgae in the present study.

Using laboratory experiments, we investigated the utilization of inorganic and organic N (NO_3_^−^, NH_4_^+^, urea, and glycine) and P (PO_4_^3−^, ATP, and G-6-P) by *U. prolifera* and their growth rates in different nutrient treatments, and to explore the idea that dissolved organic nutrients may be important in supporting the *in situ* growth of *U. prolifera*. The results of these experiments could provide indications on factors responsible for the occurrence of macroalgal blooms in the southern Yellow Sea.

## Results

### General results of the ANOVAs

We conducted time-course experiments with four N- and three P-substrates, where we measured changes in the biomass (measured as fresh weight) of *U. prolifera*, the relative growth rates of algae (*K*_*i*_) and their uptake rates (*V*) of the various substrates. In the six two-way ANOVAs on the results of these experiments (Supplementary Tables S1–S6), there was no significant interaction (α = 0.05) between the two factors, i.e. the duration of incubations and the substrate type (*P* = 0.10, 0.56 and 0.29, respectively, for the N experiments; *P* = 0.22, 0.62 and 0.32, respectively, for the P experiments).

There was a significant effect (α = 0.05) of the N-substrate in the two-way ANOVAs conducted on biomass, *K*_*i*_ and *V* (*P* < 0.001, <0.001 and <0.05, respectively) and of the P-substrate in the ANOVAs conducted on biomass and *V* (*P* < 0.001 and <0.05, respectively), i.e. the effect of the P-substrate was not significant in the ANOVAs conducted on *K*_*i*_ (*P* = 0.17) (Supplementary Tables S1–S6). In the five cases where there was a significant effect of the substrate, pairwise comparisons between nutrient substrates were computed (Supplementary Tables S1–S4 and S6), and the results were detailed below. Also, there was a significant effect (α = 0.05) of the incubation duration on biomass, *K*_*i*_ and *V* in the six ANOVAs (*P* = 0.002, 0.014 and 0.006, respectively, for the N experiments; *P* < 0.001, <0.001 and <0.01, respectively, for P experiments).

For each treatment, we computed the average (*K*_*a*_, % d^−1^) and maximum (*K*_*m*_, % d^−1^) relative growth rates of *U. prolifera* (Tables 1 and 2), determined as average and maximum values of *K*_*i*_ during the 13- and 19-day N- and P-experiments, respectively. In the four one-way ANOVAs on these values, there was a significant effect (α = 0.05) of both the N- and P-substrates (Supplementary Tables S7 and S8). In these four ANOVAs, values were significantly higher in the nutrient-enriched media than in the control group, and most of the pairwise comparisons between nutrient substrates were significant (Supplementary Tables S7 and S8), as detailed below.

### Effects of N on growth rates of *U. prolifera*

**Figure 1:**
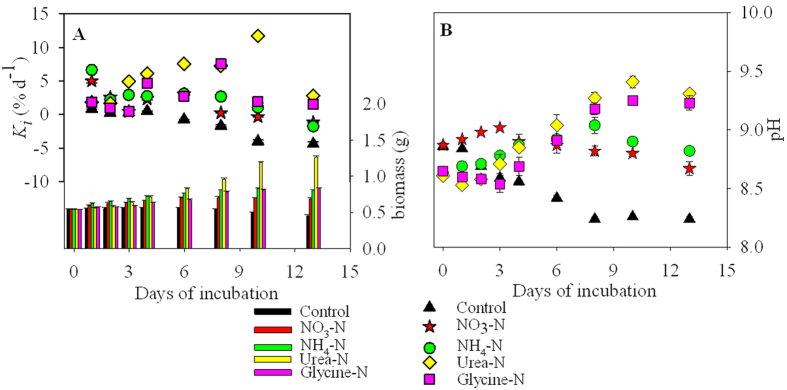
Responses of *U. prolifera* to N treatments during the 13-day experiment: changes in (**A**) biomass (histogram, right-side, *Y-axis*) and relative growth rate between successive incubation periods (*K*_*i*_, dots, left-side, *Y axis*), and (**B**) pH. Means values ± SD (all points: *n* = 3).

**Figure 2:**
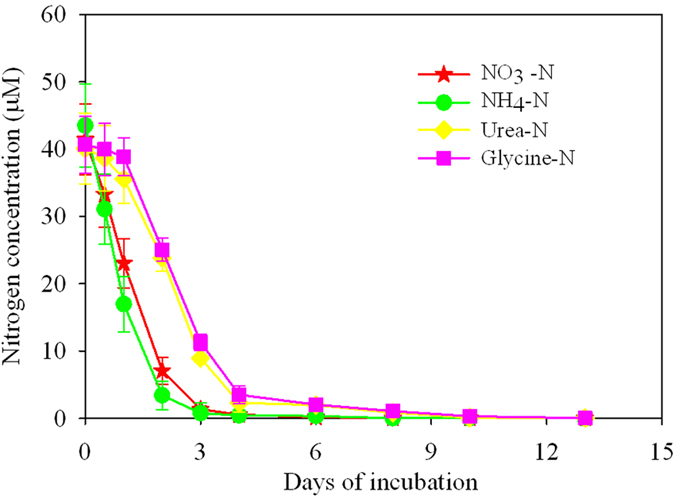
Changes in concentrations of the different substrates in the culture medium of the four N treatments during the 13-day experiment. Means values ± SD (all points: *n* = 3).

The growth responses (biomass and *K*_*i*_; Fig. 1A, Supplementary Tables S1 and S2) of *U. prolifera* during the 13-day experiments varied with incubation duration. In the N-enriched samples, there was an increase in biomass over the incubation period, and the responses of biomass production and growth rate were significantly higher when incubations were enriched with NH_4_^+^ and organic N than in the control. There were no significant differences for *U. prolifera* incubated with NO_3_^−^ and in the control. The relative growth rates of *U. prolifera* incubated with urea were significantly higher than those with NO_3_^−^. From the third day onwards, macroalgal biomass increased more in the two organic-N than the two inorganic-N treatments, with the greatest biomass and *K*_*i*_ occurring in the urea enrichment on day 10. After about 6–10 days of incubation, the *K*_*i*_ of *U. prolifera* in the NO_3_^−^ treatment remained relatively constant, whereas the *K*_*i*_ in the urea treatment continued to grow until about day 10 (Fig. 1A) after nutrients were depleted (Fig. 2). The *K*_*i*_ in the NH_4_^+^ and glycine treatments were not significantly different during the incubations (Supplementary Table S2). On day 13, *U. prolifera* incubated with urea and glycine had higher biomass compared with those incubated with NO_3_^−^ or in the absence of added nutrients.

Most values of *K*_*a*_ and *K*_*m*_ were significantly higher (α = 0.05) in the treatments with organic-N (especially urea) than inorganic-N, but there was no significant difference in the *K*_*a*_ of the NH_4_^+^ and glycine treatments (Table 1, and Supplementary Table S7).

Generally, the pH increased during all N-enriched incubations except in the NO_3_^−^ treatment and the un-enriched control (Fig. 1B). Urea and glycice enriched incubations had the highest pH and in all treatments, the pH decreased toward the end of the experiment.

### Change in concentration of the N substrates during the experiments, and N uptake by *U. prolifera*

*Ulva prolifera* removed nearly all inorganic N by day 3 and the organic N by day 6 of the 13-day experiment (Fig. 2). By day 3, 97, 98, 78 and 72% of the NO_3_^−^, NH_4_^+^, urea and glycine, respectively, had been removed, and the concentrations of urea and glycine remaining in the culture medium were 8.5 and 11.0 μM, respectively. By day 8, the organic-N substrates were fully depleted. Moreover, the inorganic-N concentrations in the media enriched with the two organic-N substrates, i.e. urea and glycine, remained below detection throughout the experiment.

**Figure 3:**
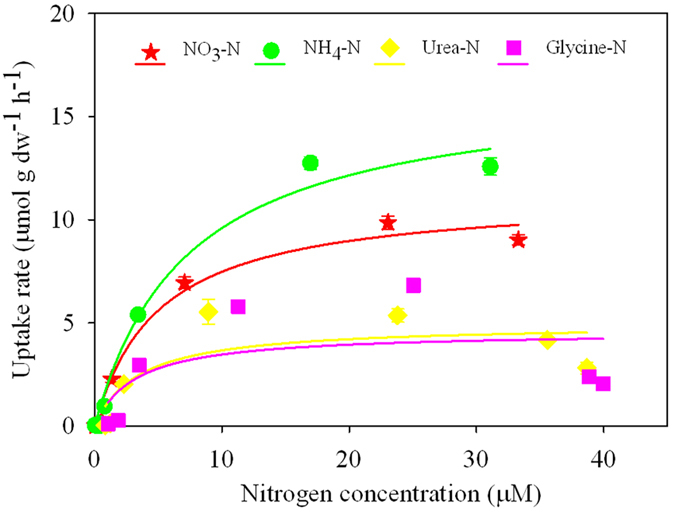
Concentration-dependent uptake rates of the four nitrogen substrates by *U. prolifera* during the first eight days (192 h) of the experiment. Dots: uptake rates.

The uptake rate and *V*_max_/*K*_*s*_ of NH_4_^+^ were the highest among the four N-substrates (Fig. 3 and Supplementary Table S9). The uptake rates of NO_3_^−^ and NH_4_^+^ were significantly higher (α = 0.05) than those of urea and glycine (Supplementary Table S3).

### Effects of P on growth rates of *U. prolifera*

**Figure 4:**
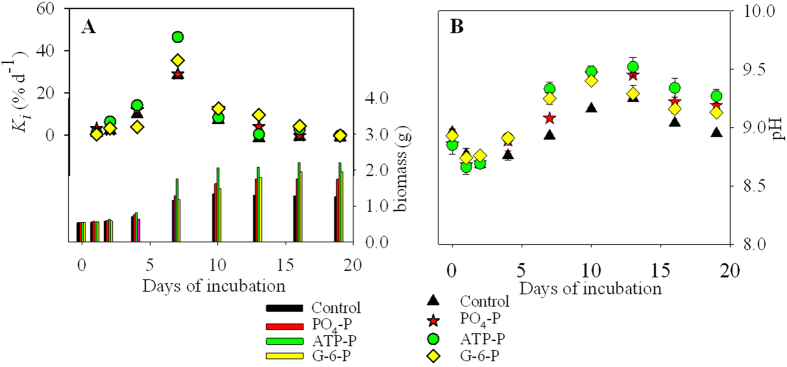
Responses of *U. prolifera* to P treatments during the 19-day experiment: changes in (**A**) biomass (histogram, right-side, *Y-axis*) and relative growth rate between successive incubation periods (*K*_*i*_, dots, left-side, *Y axis*), and (**B**) pH. Means values ± SD (all points: *n* = 3).

The growth responses (biomass and *K*_*i*_; Fig. 4A, Supplementary Tables S4 and S5) of *U. prolifera* during the 19-day experiments varied with incubation duration, and showed overall increases in biomass over the incubation period. The initial concentrations was 2.5 μM in all P-enriched samples, and there was no significant effect (α = 0.05) of the adding P on *K*_*i*_. The biomass of *U. prolifera* in samples supplemented with ATP was significantly higher than those treated with PO_4_^3−^.

The *K*_*a*_ and *K*_*m*_ were significantly higher in the treatments with organic-P (especially ATP) than PO_4_^3−^ (Table 2, and Supplementary Table S7). The biomass of the ATP-enriched cultures was consistently the highest, except on the first day of the experiment when biomass was higher in the PO_4_^3−^ treatment (Fig. 4A). From day 2 onwards, the biomass in the two organic P-treatments (ATP and G-6-P) exceeded that in the inorganic P-treatment, particularly after day 13 (Fig. 4A).

The pH in all incubations increased from about day 1 to day 13, after which they decreased in both the control and all treatments (Fig. 4B).

### Change in concentration of the P substrates during the experiments, and P uptake by *U. prolifera*

**Figure 5:**
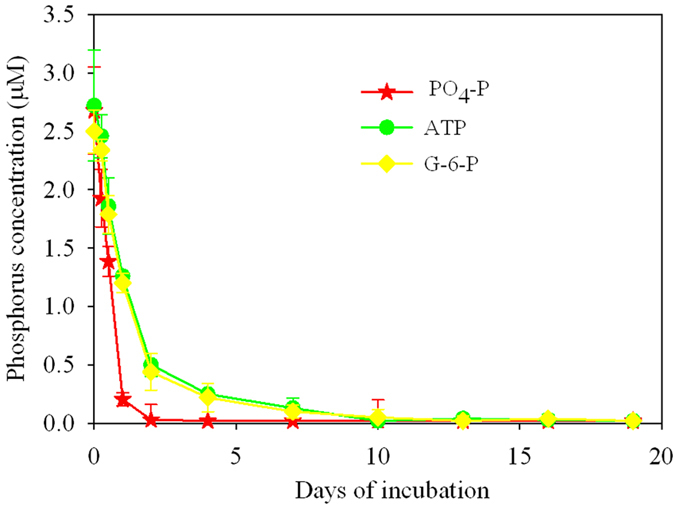
Changes in concentrations of the different substrates in the culture medium of the three P treatments during the 19-day experiment. Means values ± SD (all points: *n* = 3).

*Ulva prolifera* removed all the PO_4_^3−^ by day 2 and the organic-P by day 10 of the 19-day incubation (Fig. 5). By day 1, 92, 54 and 52% of the PO_4_^3−^, ATP and G-6-P, respectively, had been removed. The two organic substrates decreased more slowly than PO_4_^3−^, but were totally depleted on day 10. Moreover, the dissolved inorganic phosphorus concentrations in the media enriched with the two organic-P substrates, i.e. ATP and G-6-P, remained below detection throughout the experiment.

**Figure 6:**
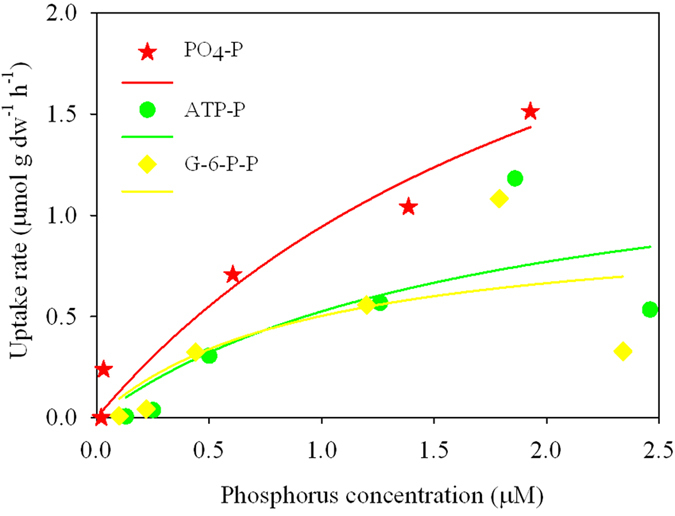
Concentration-dependent uptake rates of the three phosphorus substrates by *U. prolifera* during the first seven days (168 h) of the experiment. Dots: uptake rates.

The uptake rate of PO_4_^3−^ and the *V*_max_/*K*_*s*_were the highest among the three P-substrates (Fig. 6, and Supplementary Table S10). The uptake rate of PO_4_^3−^ was significantly higher (α = 0.05) than those of ATP and G-6-P (Supplementary Table S6).

### Effect of the changes in pH on the uptake of dissolved inorganic carbon (DIC) by *U. prolifera* in N and P experiments

The pH in the southern Yellow Sea ranged from 7.73 to 8.15, 7.99 to 8.35 and 7.98 to 8.65 in the early (11–26 April), mid (30 April–31 May), and late (13–23 June) periods of green tides in 2010, with average values of 7.85 ± 0.06, 8.07 ± 0.08, 8.14 ± 0.06, respectively^33^. In the Supplementary Material, we used the pH to calculate potential DIC uptake by *U. prolifera* at the beginning of incubations. Based on pH in the N- and P-experiments and in the field, we calculated the effect of differences in pH on the uptake of DIC by *U. prolifera* at the beginning of incubations relative to field conditions in the southern Yellow Sea. Results showed that the DIC uptake by *U. prolifera* was potentially ~10% lower in the incubation containers at the beginning of experiments than in the southern Yellow Sea. Similarly, the DIC uptake by *U. prolifera* at the beginning of the N- and P-experiments varied by ~3 and ~1%, respectively, between the highest and lowest initial pH values.

### Relationship between concentrations of organic nutrients and macroalgal blooms in the southern Yellow Sea

To examine the relationship between dissolved organic-N nutrients and macroalgal blooms in the southern Yellow Sea, we plotted the biomass of *U. prolifera* in surface water as a function of the field concentrations of urea and dissolved organic N and P, during four periods between April and June 2012 (Supplementary Fig. S2A–L). There were significant inverse correlations (Spearmann rho, α = 0.05) between macroalgal biomass with all nutrients during the 31 May–9 June 2012 period (Supplementary Fig. S2D,H,L). There were also significant inverse correlations with the urea concentrations during the 11–21 May and 25–29 May 2012 periods (Supplementary Fig. S2B,C).

## Discussion

### Methodological considerations

We examine here three methodological aspects of our results. These are the precautionary addition of nutrients in the experiments, the pH in the nutrient-amended treatments, and the possible effect of bacterial uptake on nutrient concentrations during the incubations.

There were two important differences between the results of experiments with N- and P-enrichments. Firstly, the nutrient type had a significant effect on *K*_*i*_ in the N-experiments but not in the P-experiments (Supplementary Tables S2 and S5). Secondly, the biomass in the controls did not increase during the N-experiments but did during the P-experiments (Figs 1A and 4A). In all experiments, we added 10 μM PO_4_^3−^ to the N-experiments and 100 μM NO_3_^−^ to the P-experiments, including the controls. This precautionary addition of PO_4_^3−^ to the N-experiments was done to prevent P from limiting growth during the N-amended treatments and *vise-versa.* The different responses of the controls in the two sets of experiments suggested that the NO_3_^−^ concentration added in the P-experiments stimulated *K*_*i*_ of *U. prolifera*. However, this possible methodological problem did not prevent the added P-substrate from having a significant effect on the biomass and *V* in experiments with P-treatments (Supplementary Tables S4 and S6).

Temporal variations in the pH of the growth medium reflected the growth of *U. prolifera* (Figs 1B and 4B). Indeed, increased pH likely corresponded to a decrease in dissolved CO_2_ caused by enhanced photosynthesis and biomass production due to amendment with limiting nutrients^34^. Given that there was no difference in the pH of the culture medium before addition of the N- and P- amendments, these were likely the cause of the initial pH differences among treatments (Figs 1B and 4B). The initial pH in the two sets of experiments (i.e. 8.61–8.87 and 8.86–8.96 in the N- and P-incubations, respectively) was higher that of coastal seawater in the southern Yellow Sea (approximately 8.0)^33^, but previous studies indicated that *U. prolifera* could grow well at pH between 6 and 10, and their optimal pH for the growth was 8 to 9^35^,^36^. The higher pH in the incubation bottles than in coastal seawater at the beginning of our experiments could have lead to a 10% lower DIC uptake by *U. prolifera* in the incubations than in the natural environment. It was not possible use the same approach to derive changes in DIC uptake from changes in pH during the course of the experiments because constant alkalinity cannot be assumed in our incubation containers^37^. The observed decrease in pH after maximum values in mid-experiments suggested that the cultured were probably not, or at least not severely carbon limited at the end of the experiments. The relative difference between the highest and lowest DIC uptake at the beginning of N and P experiments, derived from differences in pH, was ~3% and ~1%, respectively. Such small differences did not likely influence much carbon uptake in different experiments.

The fact that *U. prolifera* grew with both inorganic or organic N- or P-nutrients (Tables 1 and 2), and their decrease during the experiments (Figs 2 and 5) could be interpreted in at least three different ways: (1) the nutrients were taken up directly by *U. prolifera*, (2) the organic nutrients were assimilated by bacteria, and transformed by bacteria into inorganic N- or P-forms that were subsequently taken up by the macroalgae, or (3) the inorganic or organic N and P were taken up by bacteria (attached to the macroalgae or free-living), and not by *U. prolifera*. We interpreted the observed decreases in nutrient concentrations during the experiments (Figs 2 and 5) as uptake by *U. prolifera* (Figs 3 and 6) and not by attached free-living bacteria or microalgae. Bacteria and the microalgae attached to *U. prolifera* and in the culture media were killed or their numbers were largely reduced before incubation by cleaning and antibiotic treatments. Although some bacteria might have grown during the incubations, their influence on nutrient uptake was small relative to that of the macroalgae (Supplementary Fig. S3). The pretreatment of *U. prolifera* with antiobitics^38^, the subsequent washing in sterile seawater, and the use of sterile glassware and techniques throughout the experiments should have avoided introducing heterotrophic bacteria into the cultures and minimized the growth of epiphytic bacteria that may have survived the antibiotic treatment. Nevertheless, we assessed the potential uptake of nutrients by bacteria during the incubations by simulating bacterial-mediated nutrient uptake at three representative growth rates (Supplementary Material). In the modeled situation, the bacterial-mediated decline in nutrients was greater at the higher bacterial growth rates, and most of the uptake of N and P by bacteria occurred after day 6 to 12 (Supplementary Fig. S3). This was different from our observations during the incubations with *U. prolifera*, where >90% of the N and P uptake occurred by days 2 to 4 and 1 to 5, respectively (Figs 2 and 5). We concluded that the effect of bacterial uptake on the decline of N and P during the incubations with *U. prolifera* was small, and did not biased the interpretation of the experimental results.

### Growth of *U. prolifera* in experiments

The biomass and the relative growth rate of *U. prolifera* increased for all inorganic and organic N- and P-additions (Figs 1 and 4), indicating that *U. prolifera* used various N- or P-nutrient substrates for growth. The highest growth responses were observed in two of the organic N- and P-treatments, i.e. urea and ATP (*K*_*m*_, Tables 1 and 2), during the mid-to-late incubation periods (Figs 1 and 4). The combination of these laboratory results on the uptake and growth of *U. prolifera* using organic N and P with the field data of 31 May to 9 June 2012 showing significant inverse correlations between *U. prolifera* biomass and the concentrations of organic nutrients (Supplementary Fig. S2) led us to suggest that organic nutrients may have been used by *U. prolifera* for growth at sea, especially when inorganic concentrations at sea were low during the mid- to late period of the macroalgal blooms^6^,^18^.

### Uptake of inorganic and organic nutrients by *U. prolifera*

Nutrient uptake rates by macroalgae have often been described as a saturating function of substrate concentration using the Michaelis–Menten equation^39^. We used the Michaelis–Menten relationship to describe the uptake of inorganic and organic N-nutrient by *U. prolifera* (Fig. 3). The *V*_max_/*K*_*s*_ for the uptake of urea and glycine were lower than those for NO_3_^−^ and NH_4_^+^ (Supplementary Table S9), which suggested more efficient uptake of inorganic than organic N-nutrients by the *U. prolifera* fronds. We also found that the growth rate of *U. prolifera* was significantly higher in the urea and glycine and NH_4_^+^ compared to NO_3_^−^ treatment (Supplementary Table S2). This was likely due to the differences in N uptake kinetics of *U. prolifera*. *U. prolifera* had a higher affinity for NH_4_^+^ than NO_3_^−^ in seawater^16^,^39^, which was owing to the lower amount of energy required for assimilation of NH_4_^+^ than NO_3_^−^ given that NO_3_^−^ needs to be reduced to NH_4_^+^ by nitrate reductase before being assimilated^40^. Moreover, previous studies have shown that some organic-N compounds, especially organic-N released at the decline of the spring phytoplankton bloom, were likely more easily assimilated by macroalgae than NO_3_^−^ 41,42,43.

Previous laboratory experiments have shown that *U. prolifera* take up N at a markedly higher *V*_max_/*K*_*s*_ than P^16^, and our present results show that *U. prolifera* can take up both N- and P-nutrients, including some forms of organic-P (ATP and G-6-P, Fig. 6; Supplementary Table S10). In the southern Yellow Sea from April to June 2012, the concentrations of PO_4_-P and organic-P ranged from undetectable to 0.6 μM and from undetectable to 1.2 μM, respectively, and the organic-P concentrations can account for ~65% of the total dissolved phosphorus^6^. Since the *V*_max_/*K*_*s*_ value in experiments with PO_4_^3−^ was higher than the values for ATP and G-6-P (Fig. 6, Supplementary Table S10), we concluded that *U. prolifera* preferentially assimilated inorganic- over organic-P substrates.

Our laboratory results showed a significant (α = 0.05) increase in *U. prolifera* biomass when enriched with ATP and G-6-P, but not in treatments enriched with PO_4_^3−^ (Supplementary Table S4, control vs. treatments). Previous investigations had shown that P-limited phytoplankton could take up labile and low molecular weight organic-P from seawater especially when PO_4_^3−^ concentrations were low^25^,^44^,^45^. ATP, one of labile organic-P forms, is the powerhouse for nutrient uptake by phytoplankton as it can store and provide energy for nutrient assimilation^46^,^47^. We hypothesized that in our experiment the significant increase in fresh weight of *U. prolifera* under treatments enriched with organic-P may result in part from the enhanced N uptake driven by organic-P addition in the medium^45^.

Among the most important findings of our laboratory experiments was that the bloom-forming macroalga *U. prolifera* took up both inorganic and organic nutrients, supporting the idea that organic nutrients may contribute to the development of green macroalgal blooms in the southern Yellow Sea.

## Conclusion

During the nine successive years of massive blooms of green macroalgae in the southern Yellow Sea, these blooms always started in coastal waters of the Jiangsu Province during mid-April. The present study provides experimental evidence that is consistent the idea that organic nutrients may be involved in the occurrence of green-tide blooms in the southern Yellow Sea. The role of organic nutrients could be particularly important when inorganic nutrients are low, i.e. during the mid to late period of the annual development of the green-tide blooms^18^. This suggests that controlling the discharge of organic nutrients, such as unused feed and organic excreta from rivers and mariculture ponds, may reduce the annual occurrence of harmful macroalgal blooms in the southern Yellow Sea.

## Materials and Methods

### Algal material collection and pre-treatment

Fronds of *U. prolifera* were sampled from *Porphyra yezoensis* mariculture rafts in Rudong (121°5′20.4″N; 32°40′51.6″E), Jiangsu Province, China, in April 2013. The intact and healthy algal fronds collected *in situ* were stored at approximately 4 °C for transport to the laboratory (within 48 h). Upon arrival, the fronds were gently washed several times with sterile seawater (autoclave, 120 °C, 20 min), cleaned with 1% sodium hypochlorite for 1–2 min, and rinsed with sterile artificial seawater. Epiphytes and visible particles attached to the fronds were removed, after which the fronds were placed overnight in an incubator (at 15 °C, in darkness) in sterile artificial seawater with 1 ml L^−1^ of GeO_2_ dissolved in milli-Q water (0.5 mg mL^−1^) to inhibit the growth of diatoms, and antibiotics (4% Kanamycin) to kill bacteria. The seaweeds were then incubated (in a GXZ-380B illumination incubators, Ningbo, Zhejiang, China, at 15 °C, under an irradiance of 80 μmol photons m^−2 ^s^−1^ with a 12:12 h light:dark cycle) in sterile artificial seawater (ASW) enriched with f/2 medium during 2 days^48^. Four days before the experiments, the *U. prolifera* fronds were incubated in aged ASW (salinity of 35.0, pH of 8.1) supplemented with modified f/2 medium (no N, P or Si and supplemented with trace elements and vitamins) to decrease the N or P content in *U. prolifera* tissues. The incubation containers were washed with 20% sulfuric acid (H_2_SO_4_), rinsed several times with Milli-Q water (pH = 7.0), sterilized (autoclave, 120 °C, 20 min), and dried in a muffle furnace at 450 °C during 4 h to prevent nutrient contamination. They were closed with Parafilm™, and incubated as explained above.

### Experimental design

We investigated the effects of N and P substrates on the growth and uptake rate of *U. prolifera*, during laboratory incubations. *Ulva prolifera* were incubated in 3-L incubation containers containing 1800 mL media (aged ASW + modified f/2 medium; see above). The initial algal biomass (fresh weight) in each incubation container was 0.3 g L^−1^. All experiments were conducted under the same clean but not sterile conditions, described above. The incubation containers were sealed with Parafilm™, which was briefly opened at the times of sampling. Three independent, parallel incubations (triplicates) were performed for each control and nutrient treatment. The pH of incubated samples was measured with a handheld pH meter (PB-10, Sartotius, USA; precision ± 0.01 pH unit). The pH of the medium was the same in all incubation containers before adding nutrients. The initial (day 0) pH of each treatment was measured after adding the N or P substrates and the *U. prolifera* fronds to the medium. Water samples for nutrient analyses were filtered on GF/F filters, which had been pretreated by heating at 450 °C for 4 hours, and 5 ml were stored in polyethylene flasks at −20 °C. Before taking the water samples, the algal frond inside each incubation container was collected and drained to remove excess water, and its biomass (determined as fresh weight) was determined immediately. On the last day of incubations, after measuring their wet-weight biomass, the dry weight of macroalgal tissues was determined by drying the incubated fronds at 55 °C for 72 h.

The growth of *U. prolifera* was measured as a function of added dissolved inorganic and organic N and P. The four N substrates were NO_3_^−^ (NaNO_3_), NH_4_^+^ (NH_4_Cl), urea (CO(NH_2_)_2_), and glycine (C_2_H_5_NO_2_). In each treatment, a single N substrate was added to N-depleted sterile ASW, and then N concentration was adjusted to 40 μM. The control group consisted of incubations without N addition. The treatments and control received 10 μM PO_4_^3−^ (KH_2_PO_4_) to prevent P limitation of macroalgal growth during the experiment^39^,^49^. To determine N concentrations, 5 mL of incubation medium were collected on days 0, 0.5, 1, 2, 3, 4, 6, 8, 10 and 13. The P-dependent growth of on *U. prolifera* was measured for the three PO_4_^3−^ (NaH_2_PO_4_), ATP (C_10_H_14_N_5_Na_2_O_13_P_3_), and G-6-P (C_6_H_12_NaO_9_P). In each treatment, a single P substrate was added to P-depleted sterile ASW, and the P concentration was adjusted to 2.5 μM. The control group consisted of incubations without P addition. The control and treatments receive 100 μM of NO_3_^−^ to prevent N limitation of macroalgal growth during the experiment^39^,^49^. To determine P concentrations, 5 mL of incubation medium were collected on days 0, 0.25, 0.5, 1, 2, 4, 7, 10, 13, 16 and 19.

### Sample analysis

Spectrophotometric analysis of NO_3_-N, NH_4_-N and PO_4_-P was performed using an AutoAnalyzer (BRAN and LUEBBE AA3, Germany) after the water samples had been thawed to room temperature. Total dissolved phosphorus (TDP) was measured by persulfate oxidation^50^, and dissolved organic phosphorus was obtained by subtracting dissolved inorganic phosphorus from TDP. The analytical precision of the NO_3_-N, NH_4_-N, PO_4_-P and TDP determinations were 0.04, 0.03, 0.02 and 0.02 μM, respectively. Urea concentrations in the samples were determined manually based on the reaction of urea with diacetylmonoxime, and the analytical precision was 0.03 μg at urea-N 1^−1^ 51. Free amino acids were measured by pre-column derivatization high-performance liquid chromatography, and the analytical precision was 4 to 29 fmol for individual amino acids^52^.

### Calculations

To analyze the growth responses of *U. prolifera* to various inorganic and organic nutrient treatments, we calculated the relative growth rates of macroalgal fronds with the following equation^53^:


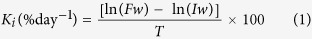


where *K*_*i*_is relative growth rate of *U. prolifera*, ln is the natural logarithm, *Fw* is the final fresh weight (i.e. at the end of the sampling interval), *Iw* is the initial fresh weight (i.e. at the beginning of the sampling interval), and *T* is the duration of the incubation interval (e.g. *T* = 3 days when the incubated water was sampled every third day).

The uptake rates for inorganic and organic N or P substrate were calculated using the observed decline in nutrient concentrations during each sampling interval with the following equation:


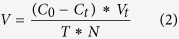


where *V* is the uptake rate of the nutrient substrate (μmol g(dw)^−1 ^h^−1^), *C*_*0*_ and *C*_*t*_ are the nutrient concentrations (μM) at the beginning and at the end of the sampling interval, respectively, *V*_*t*_ is the water volume (L) at the end of the sampling interval, *T* is the duration (h) of the sampling interval, and *N* is the dry weight (g) of macroalgae. Since the life cycle of macroalgae, such as *U.prolifera*, is longer than that of phytoplankton^54^,^55^, we used in this study longer incubation times than are typical of microalgal nutrient uptake experiments. Nitrogen and phosphorus uptake by *U. prolifera* was estimated from the decline in ambient nitrogen between incubations days 0, 0.5, 1, 2, 3, 4, 6 and 8 (i.e. 0, 12, 24, 48, 72, 96, 144, and 192 h) and 0, 0.25, 0.5, 1, 2, 4 and 7 (i.e. 0, 6, 12, 24, 48, 96, 168 h), respectively.

The Michaelis–Menten equation, originally developed to describe enzyme kinetics, has been used to describe the nutrient uptake and growth response of macroalgae^39^:


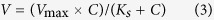


where *V* is the uptake rate of the nutrient substrate computed for each sampling interval (eq. 2) between 0 and 192 h and 0 and 168 h for the N- and P-experiments, respectively, and *C* is the nutrient concentration (μM) measured at the end of the sampling interval. In equation (3), *V*_max_ and *K*_*s*_ are the maximum uptake rate and the substrate concentration at which uptake proceeds at half the maximum rate (half-saturation constant), respectively. The kinetic parameters *V*_max_ and *K*_*s*_ were obtained for each N and P substrate by fitting equation (3) to hyperbolic tangent plots of uptake rates versus nutrient concentrations using SigmaPlot 12.5. The ratio *V*_max_/*K*_*s*_has been used an index of affinity, were high values imply high substrate affinity^56^.

### Field data from the southern Yellow Sea

We used published and unpublished field data on urea, organic-N, organic-P and *U. prolifera* biomass to determine the relationship between macroalgal blooms and nutrients at sea. These data were from four cruises conducted in coastal waters of the southern Yellow Sea (32–36° N, 120–124° E) between 27 April and 9 June 2012. The urea data are reported for the first time in the present study, whereas the other data had been reported in two previous studies, i.e. organic-N and organic-P in one study^6^ and *U. prolifera* biomass in another study^8^. The relationships between biomass and the three nutrients are analyzed here for the first time.

### Statistical analysis

ANOVA was used to assess the effect of different N and P treatments on the growth responses and nutrient uptake rates of *U. prolifera*. Firstly, biomass, *K*_*i*_ and *V* were analyzed using two-way ANOVA, where the two factors were incubation duration and the nutrient treatment. In cases where there were both no significant interaction between the two factors and a significant effect of the nutrient treatment, *a posteriori* comparisons between pairs of nutrient treatments were conducted to identify which of the treatments differed significantly from others (Holm-Sidak tests). Secondly, *K*_a_ and *K*_m_ were analyzed using one-way ANOVA, where the factor was the nutrient treatment. In cases where there was a significant effect of the nutrient treatment, *a posteriori* comparisons between pairs of nutrient treatments were conducted as done in two-way ANOVA.

Assumptions of homogeneity of variance and normality in the ANOVAs were assessed by scatter plots of residuals and normal curves of residuals, respectively (Holm-Sidak test, SigmaPlot 12.5). Spearman *rho* correlation was used to assess if there was a significant monotonic relationship between pairs of variables. We used Spearman *rho* correlation because it does not assume linear relationships between pairs of variables. Statistical analyses were performed using SigmaPlot 12.5. The significance level was α = 0.05 for all tests unless otherwise stated.

## Additional Information

**How to cite this article**: Li, H. *et al*. Growth responses of *Ulva prolifera* to inorganic and organic nutrients: Implications for macroalgal blooms in the southern Yellow Sea, China. *Sci. Rep.* **6**, 26498; doi: 10.1038/srep26498 (2016).

## Supplementary Material

Supplementary Information

## Figures and Tables

**Table 1:** Average (*K*_*a*_) and maximum (*K*_*m*_) relative growth rates (means ± SD, *n* = 3) of *U. prolifera* during the 13-day incubations with different N substrates.

**N treatments**	***K***_***a***_ (**% d**^**−1**^)	***K***_***m***_ (**% d**^**−1**^)
Control	−0.7 ± 0.04	1.7 ± 0.02
NO_3_^−^	2.8 ± 0.03	7.6 ± 0.14
NH_4_^+^	4.0 ± 0.11	8.1 ± 0.31
Urea	7.0 ± 0.13	11.1 ± 0.10
Glycine	4.0 ± 0.10	9.7 ± 0.03

The values of *K* used to determine *K*_*a*_ and *K*_*m*_ for each nutrient treatment and the control were computed for each of the three incubations.

**Table 2:** Average (*K*_*a*_) and maximum (*K*_*m*_) relative growth rates (means ± SD, *n* = 3) of *U. prolifera* during the 19-day incubation with different P substrates.

**P treatments**	***K***_***a***_ (**% d**^**−1**^)	***K***_***m***_ (**% d**^**−1**^)
Control	4.1 ± 0.01	12.4 ± 0.23
PO_4_^3−^	5.9 ± 0.10	15.6 ± 0.11
ATP	7.6 ± 0.02	25.4 ± 0.02
G-6-P	6.1 ± 0.02	20.2 ± 0.13

The growth rates (*K*) were computed for each of the three incubations for each nutrient treatment and control.
