# Supplementary material for: Growth responses of Ulva prolifera to inorganic and organic nutrients: Implications for macroalgal blooms in the southern Yellow Sea, China
Source: Sci Rep. 2016 May 20;6:26498. doi: 10.1038/srep26498 (PMC4873802; doi:10.1038/srep26498)
Supplement: Supplementary Information [file srep26498-s1.pdf]

Growth responses of *Ulva prolifera* to inorganic and organic nutrients: Implications for macroalgal blooms in the southern Yellow Sea, China

Hongmei Li [a], Yongyu Zhang [a *], Xiurong Han [b], Xiaoyong Shi [b,c *], Richard B. Rivkin [d], Louis Legendre [e]

[a] Research Center for Marine Biology and Carbon Sequestration, Shandong Provincial Key Laboratory of Energy Genetics, Qingdao Institute of Bioenergy and Bioprocess Technology, Chinese Academy of Sciences, Qingdao, 266101, China

[b] College of Chemistry and Chemical Engineering, Ocean University of China, 238 Songling Road, Qingdao 266100, PR China

[c] National Marine Hazard Mitigation Service, 6 Wangfen North Road, Beijing 100194, PR China

[d] Department of Ocean Sciences, Memorial University of Newfoundland, St. John's, NL A1C 5S7, Canada

[e] Sorbonne Universités, UPMC Université Paris 06, CNRS, Laboratoire d'océanographie de Villefranche (LOV), Observatoire océanologique, 181 Chemin du Lazaret, 06230 Villefranche-sur-Mer, France

SUPPLEMENTARY MATERIAL

Submitted to《Scientific Reports》

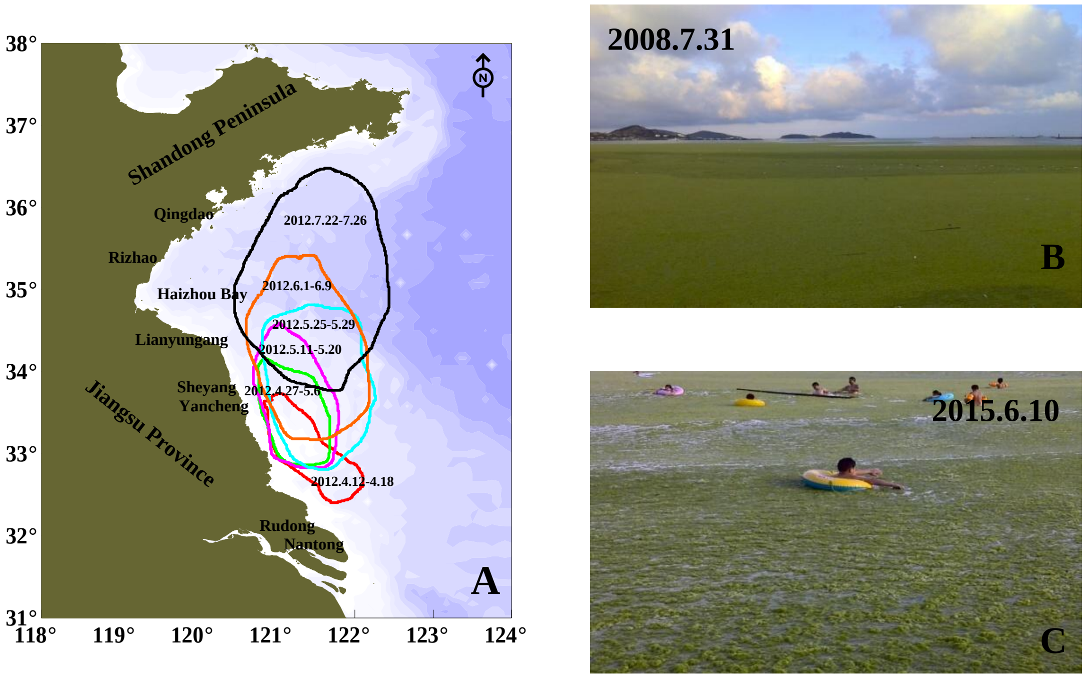

Fig. S1. Green tide blooms in the southern Yellow Sea, China. (A) Map of the Yellow Sea. Different colors represent the path of increasing large floating green tides from south to north during the spring and summer of 2012. (B and C) Pictures of the massive macroalgal blooms in the southern Yellow Sea in 2008 and 2015, respectively. Panel A was generated using Surfer 8.0. (http://www.goldensoftware.com).

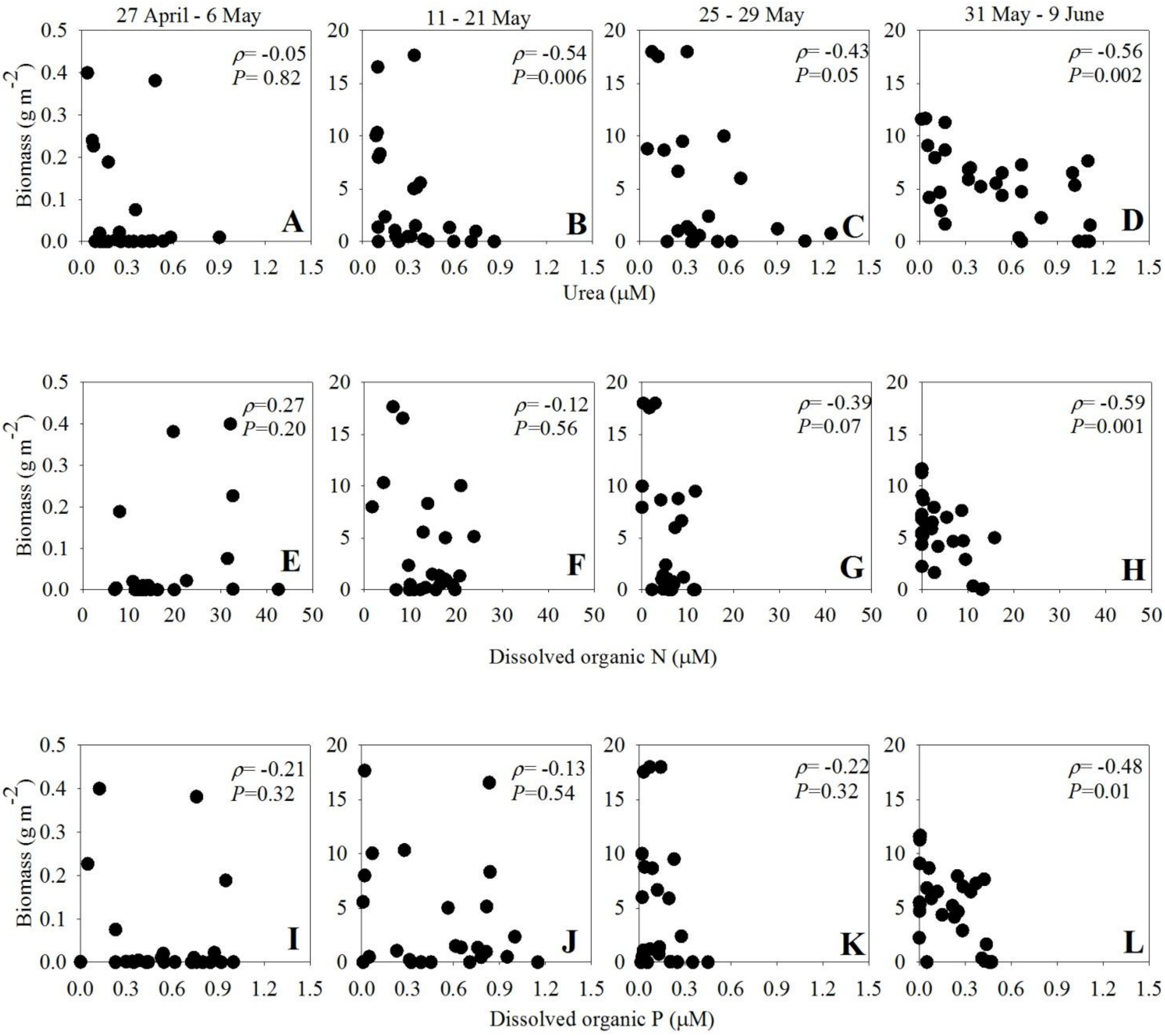

Fig. S2. Biomass of *U. prolifera* in surface water during the occurrence of macroalgal blooms as a function of concentrations of dissolved organic nutrients (urea, DON and DOP). All samples were collected in coastal surface water (0–5 m) of the southern Yellow Sea during four cruises between 27 April and 9 June 2012.The *U. Prolifera* biomass data were presented in Ref S1and the DON and DOP data in Ref S2.

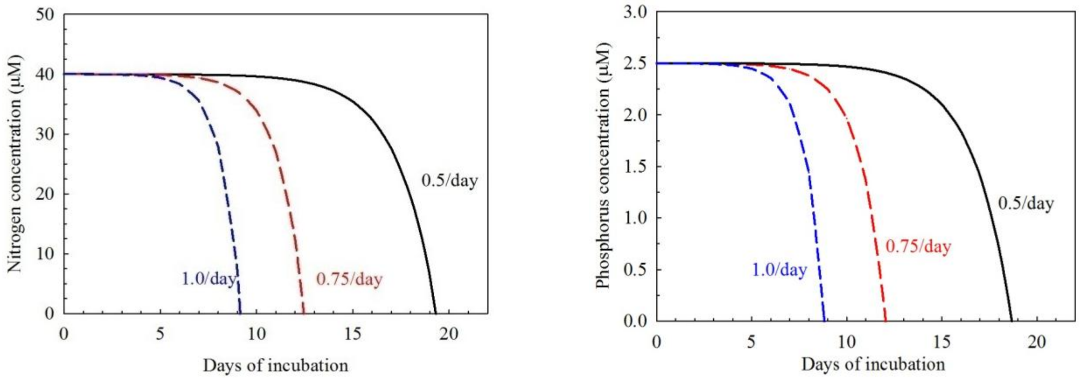

Fig. S3. Modeled bacterial-mediated decline in N and P concentrations in the incubation medium for bacteria growing at 0.5, 0.75 and 1.0 divisions per day. Assumptions are that the initial abundance of bacteria is 33000 cells L^{-1}, a carbon content of 1.25 fmol $cell^{-1}$ (i.e. 15 fg $cell^{-1}$), and a C:N = 5 and C:P = 25.

Table S1

Results of two-way ANOVA on biomass of *U. prolifera* in the different N-treatments, and of a *posteriori* pairwise multiple comparisons (Holm-Sidak test) for factor 2 (N-substrate).

**Sources of variation**	***P***
Factor 1 (Incubation duration)	0.002
Factor 2 (N-substrate)	< 0.001
Interaction (factor 1 x factor 2)	0.10
***A posteriori* pairwise comparisons**	***P***
Control vs. NO_3^-	0.215
Control vs. NH_4^+	0.044
Control vs. Urea	< 0.001
Control vs. Glycine	0.037
NO_3^- vs. NH_4^+	0.723
NO_3^- vs. Urea	0.040
NO_3^- vs. Glycine	0.596
NH_4^+ vs. Urea	0.177
NH_4^+ vs. Glycine	0.975
Urea vs. Glycine	0.239

Table S2

Results of two-way ANOVA on relative growth rates (K_i) in the different N-treatments, and of a *posteriori* pairwise multiple comparisons (Holm-Sidak test) for factor 2 (N-substrate).

**Sources of variation**	***P***
Factor 1 (Incubation duration)	0.014
Factor 2 (N-substrate)	< 0.001
Interaction (factor 1 x factor 2)	0. 56
***A posteriori* pairwise comparisons**	***P***
Control vs. NO_3^-	0.059
Control vs. NH_4^+	0.008
Control vs. Urea	< 0.001
Control vs. Glycine	0.007
NO_3^- vs. NH_4^+	0.714
NO_3^- vs. Urea	0.021
NO_3^- vs. Glycine	0.576
NH_4^+ vs. Urea	0.116
NH_4^+ vs. Glycine	0.988
Urea vs. Glycine	0.139

Table S3

Results of two-way ANOVA on nutrient uptake rates (*V*) in the different N-treatments, and of a *posteriori* pairwise multiple comparisons (Holm-Sidak test) for factor 2 (N-substrate).

**Sources of variation**	***P***
Factor 1 (Incubation duration)	0.006
Factor 2 (N-substrate)	0.046
Interaction (factor 1 x factor 2)	0.29
***A posteriori* pairwise comparisons**	***P***
NO_3^- vs. NH_4^+	0.521
NO_3^- vs. Urea	0.047
NO_3^- vs. Glycine	0.045
NH_4^+ vs. Urea	0.030
NH_4^+ vs. Glycine	0.021
Urea vs. Glycine	0.850

Table S4

Results of two-way ANOVA on biomass of *U. prolifera* in the different P-treatments, and of a *posteriori* pairwise multiple comparisons (Holm-Sidak test) for factor 2 (P-substrate).

**Sources of variation**	***P***
Factor 1 (Incubation duration)	< 0.001
Factor 2 (P-substrate)	< 0.001
Interaction (factor 1 x factor 2)	0.22
***A posteriori* pairwise comparisons**	***P***
Control vs. PO_4^{3-}	0.058
Control vs. ATP	< 0.001
Control vs. G-6-P	0.030
PO_4^{3-} vs. ATP	0.030
PO_4^{3-} vs. G-6-P	0.551
ATP vs. G-6-P	0.072

Table S5

Results of two-way ANOVA on relative growth rates (K_i) in the different P-treatments. Because the effect of the P-substrate in the ANOVA was not significant, pairwise multiple comparisons between the P-treatments were not conducted.

**Sources of variation**	***P***
Factor 1 (Incubation duration)	< 0.001
Factor 2 (P-substrate)	0.17
Interaction (factor 1 x factor 2)	0. 62

Table S6

Results of two-way ANOVA on nutrient uptake rates (*V*) in the different P-treatments, and of a *posteriori* pairwise multiple comparisons (Holm-Sidak test) for factor 2 (P-substrate).

**Sources of variation**	***P***
Factor 1 (Incubation duration)	0.007
Factor 2 (P-substrate)	0.049
Interaction (factor 1 x factor 2)	0.32
***A posteriori* pairwise comparisons**	***P***
PO_4^{3-} vs. ATP	0.045
PO_4^{3-} vs. G-6-P	0.040
ATP vs. G-6-P	0.715

Table S7

Results of one-way ANOVA on average (K_a) and maximum (K_m) relative growth rates in the different N-treatments, and of a *posteriori* pairwise multiple comparisons for the N-substrate (Holm-Sidak test).

**Sources of variation**	$P_{(Ka)}$	$P_{(Km)}$
Factor (N-substrate)	< 0.001	< 0.001
***A posteriori* pairwise comparisons**	$P_{(Ka)}$	$P_{(Km)}$
Control vs. NO_3^-	< 0.001	< 0.001
Control vs. NH_4^+	< 0.001	< 0.001
Control vs. Urea	< 0.001	< 0.001
Control vs. Glycine	< 0.001	< 0.001
NO_3^- vs. NH_4^+	< 0.001	< 0.001
NO_3^- vs. Urea	< 0.001	< 0.001
NO_3^- vs. Glycine	< 0.001	< 0.001
NH_4^+ vs. Urea	< 0.001	< 0.001
NH_4^+ vs. Glycine	0.372	< 0.001
Urea vs. Glycine	< 0.001	< 0.001

Table S8

Results of one-way ANOVA on average (K_a) and maximum (K_m) relative growth rates in the different P-treatments, and of a *posteriori* pairwise multiple comparisons for the P-substrate (Holm-Sidak test).

**Sources of variation**	$P_{(Ka)}$	$P_{(Km)}$
Factor (P-substrate)	< 0.001	< 0.001
***A posteriori* pairwise comparisons**	$P_{(Ka)}$	$P_{(Km)}$
Control vs. PO_4^{3-}	< 0.001	< 0.001
Control vs. ATP	< 0.001	< 0.001
Control vs. G-6-P	< 0.001	< 0.001
PO_4^{3-} vs. ATP	< 0.001	< 0.001
PO_4^{3-} vs. G-6-P	0.02	< 0.001
ATP vs. G-6-P	< 0.001	< 0.001

Table S9

Uptake kinetic parameters of *U. prolifera* in the different N-treatments. Values are means ± SD (n = 3).

Nutrient treatment	V_{max} (μmol g(dw)$^{-1}$ h^{-1})	K_s (μM)	V_{max}/K_s
Nitrate (NO_3^-)	11.2 ± 0.6	5.0 ± 0.6	2.2
Ammonium (NH_4^+)	16.6 ± 0.7	7.3 ± 0.5	2.3
Urea	4.9 ± 0.7	3.5 ± 0.3	1.4
Glycine	4.6 ± 0.2	3.2 ± 0.8	1.4

Table S10

Uptake kinetic parameters of *U. prolifera* in the different P-treatments. Values are means ± SD (n = 3).

P treatment	V_{max} (μmol g(dw)$^{-1}$ h^{-1})	K_s (μM)	V_{max}/K_s
PO_4^{3-}	3.3 ± 0.2	2.5 ± 0.1	1.3
ATP	1.4 ± 0.1	1.8 ± 0.5	0.8
G-6-P	1.0 ± 0.2	1.0 ± 0.14	1.0

Effects of pH on DIC uptake by *U. prolifera*

We estimated the potential effect of differences in pH on the uptake of dissolved inorganic carbon (DIC) by *U. prolifera* at the beginning of incubations relative to field conditions. Based on the pH values measured in the present study and in the southern Yellow Sea in 2010[S3] our calculations were as follows:

(1) The approximate field pH in the southern Yellow Sea during the macroalgal blooms of 2010 was ~8.0 (pH_{SYS}), and the pH at the beginning of our incubations was ~8.8 (pH_{incub}). Hence:

$$\Delta pH = pH_{SYS} - pH_{incub} = 0.8 \qquad (S1)$$

(2) We calculated the relative difference in DIC uptake (ΔDIC) by *U. prolifera* by combining equations (1) and (2) in the Appendix of Axelsson (1988)[S4] assuming that alkalinity was the same in our incubation bottles at the beginning of incubations as in the field. Our equation was:

$$\Delta DIC / DIC_{SYS} = \Delta pH / pH_{SYS} \qquad (S2)$$

$$\Delta DIC / DIC_{SYS} = 0.8 / 8.0 = 0.1 \qquad (S3)$$

The DIC uptake by *U. prolifera* was thus potentially ~10% lower in our incubation bottles at the beginning incubations than in the southern Yellow Sea.

We estimated similarly the potential effect on DIC uptake of differences in initial pH among the different N and P treatments:

(1) The highest and lowest pH values (pH_H and pH_L, respectively) among the different N-treatments were 8.87 and 8.61, respectively. Hence:

$$\Delta pH = pH_H - pH_L = 0.3. \qquad (S4)$$

(2) The average value of initial pH (pH_{AVE}) for the different N-treatments was [8.87+8.61)/2] = 8.75. Hence:

$$\Delta DIC / DIC_{AVE} = \Delta pH / pH_{AVE} = 0.3 / 8.75 = 0.03 \qquad (S5)$$

The relative difference between the highest and lowest DIC uptake at the beginning of the N-experiment, derived from differences in pH, was thus ~3%. Using the same approach, we found that the corresponding value for the P-experiment was ~1%.

Modeling the potential uptake of nutrients by bacteria during the incubations

We assessed the potential uptake of nutrients by bacteria during the incubations by numerically simulating their growth and nutrient uptake. In the natural environment, the average density of epiphytic bacteria on macroalgae ranges from 10^4 to 5×10^5 cells per gram of algal fresh weight[S5]. The initial fresh-weight biomass of *U. prolifera* at the start of the experiments was ~0.3 g, and assuming an average bacterial density of 7×10^4 cells per gram of algal fresh weight and an incubation volume of 1.8 L, the average bacterial abundance would have been ~33,000 cells L^{-1}. This value was an overestimate as it assumed that epiphytic bacteria survived the antibiotic treatment.

We computed the growth rate using an exponential growth model[S6]:

$$\mathbf{BA_{t2}} = \mathbf{BA_{t1}} \times e^{(kt)} \qquad (S6)$$

where $\mathbf{BA_{t2}}$ is the volume specific bacterial abundance (**BA**, cells L^{-1}) at time 2, $\mathbf{B}A_{\mathbf{t1}}$ is the volume specific bacterial abundance at time 1, **k** is the growth rate (d^{-1}) and **t** = (**t2** – **t1**) is the time interval in days. We ran the simulation, with successive one-day time steps, for 13 d and 19 d for N and P, respectively, for **k** = 0.5, 0.75 and 1.0 divisions per day (d^{-1}).

The amount of carbon fixed by bacteria during each daily time interval (**CP**, µM C) was computed as the product of the change in **BA** and an average bacterial cell carbon (**BCC**) of 1.25 fmol cell^{-1} (i.e. 15 fg cell^{-1}) [S7,S8]:

$$\mathbf{CP_{t2\text{-}t1}} = (\mathbf{BA_{t2}} - \mathbf{BA_{1}}) \times \mathbf{BCC} \qquad \text{(S7)}$$

where $\mathbf{BA_0}$ = 33,000 cells L^{-1}. The amounts of nitrogen and phosphorus taken up by bacteria during each daily time interval (**NU** and **PU**, µM N and µM P, respectively) were estimated as:

$$\mathbf{NU_{t2\text{-}t1}} = \mathbf{CP_{t2\text{-}t1}} / (\mathbf{C{:}N}) \qquad \text{(S8)}$$

$$\mathbf{PU_{t2\text{-}t1}} = \mathbf{CP_{t2\text{-}t1}} / (\mathbf{C{:}P}) \qquad \text{(S9)}$$

where the C:N and C:P ratios are 5 and 25, respectively[S9,S10,S11].

We computed the N and P concentrations in the incubation medium at the end of each time step (**t2**) as the concentration of N or P in the incubation medium at the beginning of the time step (**t2**) minus the **NU** or **PU**, respectively:

$$\mathbf{N_{t2}} = \mathbf{N_{t1}} - \mathbf{NU_{t2\text{-}t1}} \qquad \text{(S10)}$$

$$\mathbf{P_{t2}} = \mathbf{P_{t1}} - \mathbf{NU_{t2\text{-}t1}} \qquad \text{(S11)}$$

where $\mathbf{N_0}$ = 40 µM, and $\mathbf{P_0}$ = 2.5 µM (Fig. S3).

References

S1. Liu, X. Q., Li, Y., Wang, Z. L., Zhang, Q. C., Cai, X. Q. Cruise observation of *Ulva prolifera* bloom in the southern Yellow Sea, China. *Estuar. Coast. Shelf S.* **163,** 17–22 (2015).

S2. Shi, X. Y., Qi, M. Y., Tang, H. J., Han, X. R. Spatial and temporal nutrient variations in the Yellow Sea and their effects on *Ulva prolifera* blooms. *Estuar. Coast. Shelf S.* **163,** 36–43 (2015).

S3. Gao, S., Fan, S.L., Han, X.R., Li, Y., Shi, X.Y. Relations of *Ulva prolifera* blooms with temperature, salinity, dissolved oxygen and pH in the southern Yellow Sea. *China Environ. Sci.* **34**, 213–218 (2014). (in Chinese with English abstract)

S4. Axelsson, L. Changes in pH as a measure of photosynthesis by marine macroalgae. *Mar. Biol.* **97**, 287–294 (1988).

S5. Goecke, F., Labes, A., Wiese, J., Imhoff, J. F. Chemical interactions between marine macroalgae and bacteria. *Mar. Ecol. Prog. Ser.* **409**, 267–300 (2010).

S6. Kirchman, D. L. Measuring bacterial biomass production and growth rates from leucine incorporation in natural aquatic environments. In Paul, J. H. (ed.), *Method. Microbiol.* **30**, Academic Press, San Diego, pp. 227–237 (2001).

S7. Fukuda, R., Ogawa, H., Nagata, T., Koike, I. Direct determination of carbon and nitrogen contents of natural bacterial assemblages in marine environments, *Appl. Environ. Microb.* **64**, 3352–3358 (1998).

S8. Fagerbakke, K. M., Heldal, M., Norland, S. Content of carbon, nitrogen, oxygen, sulphur and phosphorus in native aquatic and cultured bacteria. *Aquat. Microb. Ecol.* **10**, 15–27 (1996).

S9. Kirchman, D. L. The uptake of inorganic nutrients by heterotrophic bacteria. *Microb. Ecol.* **28**, 255–271 (1994).

S10. Kirchman, D. L. Uptake and regeneration of inorganic nutrients by marine heterotrophic bacteria. In Kirchman, D. L. (ed.), Microbial Ecology of the Oceans. Wiley-Liss, New York, pp. 261–288 (2000).

S11. Rivkin, R. B., Anderson, M. R. Inorganic nutrient limitation of oceanic bacterioplankton. *Limnol. Oceaonogr*. **42**, 730–740 (1997).
